# Effects of the cranial parasite *Tylodelphys* sp. on the behavior and physiology of puye *Galaxias maculatus* (Jenyns, 1842)

**DOI:** 10.7717/peerj.11095

**Published:** 2021-03-22

**Authors:** Ruby López-Rodríguez, Mario George-Nascimento, Konrad Górski

**Affiliations:** 1 Programa de Magister en Ecología Marina, Universidad Católica de la Santísima Concepción, Concepción, Región del Biobío, Chile; 2 Departamento de Ecología/Facultad de Ciencias, Universidad Católica de la Santísima Concepción, Concepción, Región del Biobío, Chile; 3 Centro de Investigación en Biodiversidad y Ambientes Sustentables (CIBAS), Universidad Católica de la Santísima Concepción, Concepción, Region del Biobío, Chile; 4 Instituto de Ciencias Marinas y Limnológicas/Facultad de Ciencias, Universidad Austral de Chile, Valdivia, Región de Los Ríos, Chile

**Keywords:** Cranial cavity, Intermediate host, Diplostomatid larvae, Manipulation, Unencysted metacercariae

## Abstract

Diplostomatid digeneans are well-known manipulators of the behavior of their intermediate hosts. Unencysted metacercariae of *Tylodelphys* sp. inhabit the cranial cavity of the fish *Galaxias maculatus*; however, to date they have not been documented to alter their host behavior. The goal of this study was to evaluate the potential effects of *Tylodelphys* sp. inhabiting the cranial cavity of *Galaxias maculatus* on host physiology and swimming behavior as well as its reaction to a simulated predation attempt. Blind experiments in the lab were carried out on 56 fish that were filmed individually. The Fulton condition factor (K) was used as an approximation of nutritional status and a respirometry chamber was used to evaluate oxygen consumption rates of fish. Of the 56 fish, 21 were parasitized by *Tylodelphys* sp. (mean intensity = 30, range from 1 to 101). Parasitized and non-parasitized fish were similar in condition factor and oxygen consumption rates. Furthermore, the oxygen consumption rate of *G. maculatus* was not correlated with the abundance of *Tylodelphys* sp. However, parasitized fish more frequently swam close to the water surface, whereas non-parasitized fish more frequently swam at intermediate depths. When faced with a simulated predator attack, unparasitized fish showed more frequent fleeing behavior as well as a more intense post-fleeing activity. Collectively, these results suggest that *Tylodelphys* sp. inhabiting the cranial cavity of fish may alter their behavior predisposing them to predation by birds.

## Introduction

Parasites with complex life cycle use at least two host species to complete their life cycles, and need to move between one or two intermediate hosts (larval stage) to the definitive hosts (adult stage, where sexual reproduction occurs) (Olsen, 1986). Often, this occurs via passive trophic transmission but some parasites have the ability to modify the phenotype and behavior of their host to increase the probability of their transmission (Holmes & Bethel, 1972; Lafferty & Morris, 1996; Ruehle & Poulin, 2019; Stumbo & Poulin, 2016; Thomas, Adamo & Moore, 2005). As such, the parasite-increased trophic transmission hypothesis states that parasites maximize transmission success by increasing an intermediate hosts’ susceptibility to predation, using numerous strategies (Barber, Hoare & Krause, 2000; Barber & Wright, 2005; Lafferty, 1999; Moore & Gotelli, 1990). These strategies may include changes in pigmentation, swimming behavior or altered responses to stimuli (Barber, Hoare & Krause, 2000; Barber & Wright, 2005; Poulin, 1998; Poulin, 2010). Specific mechanisms that cause these changes in the intermediate host vary and range from changes due to direct presence of parasites in sensory organs (Barber, Hoare & Krause, 2000; Poulin, 2017) to the production and secretion of neuroactive compounds that can interfere with the predator-escape responses (Barber et al., 2017; Shaw et al., 2009). Furthermore, behavioral changes may also result from a parasites’ influence on nutritional status, energy efficiency, respiration, blood circulation, and locomotion (Barber, 2007; Barber & Wright, 2005). Potential changes such as oxygen consumption rates or indicators of nutritional status (e.g., Fulton’s condition factor, K) can be easily evaluated in fish (Froese, 2006). Often infected fishes appear healthy, despite showing altered behaviors that lead to increased predation by fish-eating birds (Fredensborg & Longoria, 2012; Lafferty & Morris, 1996).

The effects of diplostomid metacercariae (a larval stage of trematodes) on the behavior of their intermediate hosts have been previously studied (Alcaraz et al., 1999; Barber & Crompton, 1997; Brassard, Rau & Curtis, 1982). These metacercariae infect the eyes, heart, brain and cranial cavity of fish (second intermediate host) and subsequently are transferred via trophic transmission to bird definitive hosts and mature in their digestive tracts (Noble & Noble, 1965; Olsen, 1986). In contrast with the classic digenean life-cycle, diplostomid metacercariae are frequently unencysted and the effects of their movement on infection sites and the host are poorly understood (Blasco-Costa & Locke, 2017). Some previous studies have shown direct effects of the presence and movement of these metacercariae in the eye on the swimming behavior of the fish intermediate host (Stumbo & Poulin, 2016). Furthermore, there is some evidence of the increase of conspicuous behavior in fish that have parasites present in the brain (Fredensborg & Longoria, 2012; Lafferty & Morris, 1996) and that some changes in the fish serotonergic system may affect metabolic parameters such as oxygen uptake (Wendelaar Bonga, 1997; Winberg & Göran, 1993). Still, our knowledge of potential behavioral changes of the host in relation to the infection of the brain and the cranial cavity remains rudimentary.

This study aimed to evaluate the effect of an unencysted diplostomid cranial parasite on behavior and physiology of its fish intermediate host. Specifically, we studied *Tylodelphys* sp., a recently reported parasite that inhabits the cranial cavity of puye *Galaxias maculatus* (Jenyns, 1842) (George-Nascimento, López-Rodríguez & Górski, 2020). Puye are freshwater fish commonly found in southern South America in littoral zones of rivers and lakes where they feed (Cussac et al., 2020; Górski et al., 2018; Habit et al., 2010).

*Tylodelphys* sp. has a three-host life cycle. Species from the genus *Tylodelphys* have been found as larvae with asexual reproduction in the first intermediate host snail *Chilina dombeyana* (Bruguiére, 1789) (Flores, Brant & Loker, 2015; Flores & Semenas, 2008), as metacercariae in the second intermediate host puye *G. maculatus* (Fernández, Semenas & Viozzi, 2015; Quaggiotto & Valverde, 1992; Viozzi et al., 2009) and as adults in the final host, *Podiceps* birds (for example Lunaschi & Drago, 2004).

We hypothesized that due to its location in the fish brain and selection to favour increased predation by birds, *Tylodelphys* sp. impairs fish physiology, alters swimming behavior to increase conspicuousness, and decreases response to predation risk. Specifically, we predicted that (i) *G. maculatus* parasitized by *Tylodelphys* sp. would have lower metabolic rate (associated with decreased activity and slower movements) compared to unparasitized fish. Furthermore, we expected that parasitized fish would (ii) more frequently swim near the water surface, (iii) be characterized by reduced swimming activity, and after a simulated predation attack (iv) would less frequently escape and more frequently display freezing behavior compared to unparasitized fish.

## Materials & Methods

### Fish collection and maintenance

In November-December 2019, 56 individuals of *G. maculatus* were collected using a beach seine (5 m long, 1.5 m high and 10 mm stretched mesh size) and transported alive to the laboratory. Thirty-five individuals were collected from the Cruces River (39°33′4.83″S; 72°54′6.85″W) where *Tylodelphys* sp. has not been found (George-Nascimento, López-Rodríguez & Górski, 2020) and 21 individuals were collected from the Llacolén Lagoon (36°50′30, 11″S; 73°5′9, 73″W) where high abundance of *Tylodelphys* sp. was previously recorded. The main reason for choosing these two different locations was the contrast of the natural infection of the fish. Although the fish came from two different populations and locations, previous observations in the laboratory show there are no apparent differences in the phenotype and behavior of the fish from their populations (R. López-Rodríguez, 2019, personal observation). In the laboratory, fish from each location were placed in four 70 L aquaria and acclimated for 7 days in ambient temperature and with constant aeration. During acclimation, fish were fed daily *ad libitum* with live *Daphnia* spp. and commercial food pellets (Tubi-Cubi®, Tropical, Poland). All experiments were blind in the sense that observers did not know the population of origin and whether fish were parasitized or unparasitized until dissection.

To isolate an effect of parasitism on hosts, it is common to expose treatments and controls to parasites in the laboratory. However, because the first intermediate host is not known in Chile, we were not able to experimentally infect fish. For this reason, only naturally infected and uninfected fish were used. Because we sampled in two different sites (one known to be uninfected, one known to have mostly infected fish), differences between infected and uninfected groups could be actually due to difference between two sites rather than to parasitism itself. To control for the collection site, we applied a regression approach of parasite intensity vs host behavioral and physiological responses (see Lafferty & Morris, 1996 for a similar approach). Although working with naturally infected fish has drawbacks when it comes to assigning cause and effect relationships, it has the benefit of working with hosts that are experiencing natural infection levels.

### Physiological experiment

All experiments were performed separately with individual *G. maculatus* specimens. Fish were not fed for 5 h before the measurement started. Oxygen consumption (OC, mg O_2_ g^−1^ h^−1^) was measured using a closed flow chamber with a dissolved oxygen (DO) sensor (Q-Box Aqua®, QubitSystems, Canada). Fish were randomly chosen from the aquarium and placed in a 0.3 L respiratory chamber. Once in the chamber, each fish was left undisturbed to acclimate to the chamber with a constant flow of normoxic water for 1 h prior to the start of experiment. After acclimation, the chamber was sealed and the OC rate was calculated from the slope of the DO in water decrease curve during two hours of experimentation, except in some cases were the DO decreased below 3 mgL^−1^.

Each fish was weighed (total weight) and measured (total length) prior to the experiment. To assess the body condition of each fish, Fulton’s condition factor (K) was calculated following the equation *K* = 100 (W/L^3^), where W is the weight (g) and L is the total length (TL, cm) of the individual (Froese, 2006).

### Behavioral experiments

To assess whether the presence of *Tylodelphys* sp. alters behavior of *G. maculatus* host, we observed the same fish as in the physiological experiment individually (*n* = 56) in a glass aquarium (40 × 28 × 25 cm) covered on three sides with black plastic to reduce external stimuli during experiments. Water was completely replaced in the aquarium after each individual experiment. Each fish was placed in the test aquarium and was acclimated for 1 h and 30 min prior to the experiment (based on the “relax state” of the fish = slow operculum movements in previous trials). Then, a sports camera (Eken® H9R, Eken, China) placed 40 cm in front of the aquarium was turned on to record fish behavior. The camera position enabled a view of the whole aquarium in the recording. Behavior of each fish was evaluated by: (i) position in the water column, and (ii) their response to a simulated attack by a predatory bird.

To assess the swimming behavior within the water column, the aquarium was divided into 3 depths: surface (0–8.5 cm), middle (9.5–16.5 cm) and bottom (17.5–25 cm) in accordance with preliminary observations. Each fish was filmed for 30 min. Subsequently, the behavior of each fish was assessed in the recorded video by evaluating (i) swimming position within the water column and (ii) activity level.

Swimming position within the water column was assessed by recording the position of the fish every 3 min in a 30 min video (a total of ten records per individual). Frequencies of swimming at the surface, in the middle and at the bottom of the aquarium were calculated using these data. To assess activity level, each fish was observed three times for one minute (first minute of three five-minute intervals) in the recorded video. Subsequently, fish behavior was scored according to four categories: 0 = no activity, fish does not change position or depth; 1 = minimal activity, fish stays in the same position but changes depth a few times; 2 = moderate activity, fish changes depth and has long but slow movements; 3 = intense activity, fish moves fast and changes depth suddenly and frequently. The resulting score calculated for each fish corresponded to the sum of scores that resulted from each of the three observations and ranged between 0 and 9.

After the swimming behavior evaluation, fish were rested for 1 h in the same tank, after which the response of the fish to a simulated attack by a predatory bird was evaluated. Each fish was filmed for 1 h and was exposed to 3 simulated attacks. The simulated attack consisted of bringing a black plate close to the surface of the aquarium for approximately 3 s and producing a little disturbance in the water. The stimulus was meant to imitate the feeding of an aquatic bird. Subsequently, the primary and secondary response of each fish was analysed in recorded videos. For the primary response, after each of the three attacks, the fish was observed immediately and given a score of 1, if it escaped, or score of 0, if it did not escape. Scores were summed across the three attacks, so sum of scores was a minimum of 0 and a maximum of 3 per fish. The escape response was classified as successful (escaped) if the fish moved from the rest position. For the secondary response, the measurements began 30 s after the simulated attack (to avoid confusion with the primary response) and ended 3 min later, each fish was scored according to the following categories from the lowest to the highest reactivity: 0 = fish did not react; 1 = fish reacted and immediately froze; 2 = fish was characterized by slow and controlled movements; 3 = fish was characterized by several fast and erratic movements. The resulting score for each fish was calculated as the sum of the scores from each of the three observations, and ranged between 0 and 9.

**Figure 1:**
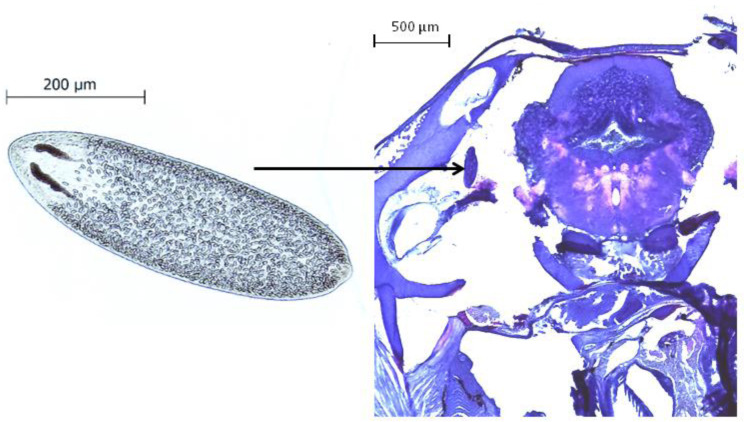
The parasite *Tylodelphys* sp. in the cranial cavity of *Galaxias maculatus*. Micrograph of *Tylodelphys* sp. individual and cross-section through the head of *Galaxias maculatus* stained with hematoxilin-eosine staining. Arrow indicates location of *Tylodelphys* sp. individual.

All behavioral videos were recorded and analysed by the same person, to whom the infection status and the source population of the observed fish was unknown. After the experimental procedure, fish were sacrificed with an overdose of 0.02% benzocaine (BZ-20®, Veterquímica, Chile) and dissected. The cranial cavity of each fish was opened under a stereomicroscope, the brain tissue was removed and placed in a Petri dish to quantify the presence and intensity of *Tylodelphys* sp. parasites (Fig. 1).

### Statistical analysis

The possible effect of *Tylodelphys* sp. on the physiology of fish was assessed by comparing the Fulton’s condition factor (*K*) and the OC rates (mg O_2_ g^−1^ h^−1^) between parasitized and unparasitized individuals using a Mann–Whitney test. Subsequently, a one-tailed non-parametric regression Theil test was used to evaluate if there was a negative slope between the intensity of parasites and the OC rate (Hollander & Wolfe, 1973). Also, a Spearman correlation used to examine the relation between infection intensity and the condition factor (K). Finally, an ANCOVA was performed to assess the differences of the OC rates between parasitized and unparasitized fish with the Fulton’s condition factor (*K*) as the covariate. These tests were performed using the online software SAS University edition.

The differences in swimming level, activity level, as well as primary and secondary response to predator stimuli (behavioral variables) between parasitized and unparasitized fish were assessed by median tests. These tests were performed separately for each variable using the online software SAS University edition.

Subsequently, all behavioral variables and OC rates together were compared between parasitized and unparasitized fish using a permutational multivariate analysis of variance (PERMANOVA) based on the Bray-Curtis distance matrix (Anderson, 2001; McArdle & Anderson, 2001). Prior to PERMANOVA data were normalized and bootstrapped (*n* = 150 resamples). In addition, Principal Coordinate Analysis (PCoA) was used to visually display the output of first and second dimensions of bootstrapping. These analyses were performed in PRIMER statistical software version 7 (Anderson, Gorley & Clarke, 2008). The significance level of each test was fixed at *α* = 0.05.

### Ethical note

We used naturally infected *G. maculatus* for our experiments, because it is the first experimental study that considers *Tylodelphys* sp. in Chile and the complete life cycle of the parasite is not known. *Galaxias maculatus* is among the most abundant and widespread native freshwater fish in Chile and more than 30 fish can be easily caught in beach seine hauled for a distance of two to five meters. In this study we trapped and used only 56 fish. To ensure suitable conditions and avoid overcrowding, during acclimatization a maximum of 20 fish was kept in each aquarium and captivity time did not exceed 3 months. After experiments, all individuals were humanely sacrificed by overdose of benzocaine 0.02%. These methods were approved by the Universidad Católica de la Santísima Concepción ethics committee and the collection method were approved by the Subsecretaría de Pesca y Acuicultura R. Ex No4060, Chilean government.

## Results

All fish caught in the Cruces River (*n* = 35) were unparasitized with *Tylodelphys* sp., whereas all fish collected in Llacolén Lagoon (*n* = 21) were parasitized with this cranial parasite. A total of 625 *Tylodelphys* sp. parasite specimens were collected from the parasitized fish and the intensity of parasites varied between 1 and 101 (mean intensity = 29.9). In addition, 5 other metazoan endoparasites taxa were recorded (Table S1).

**Figure 2:**
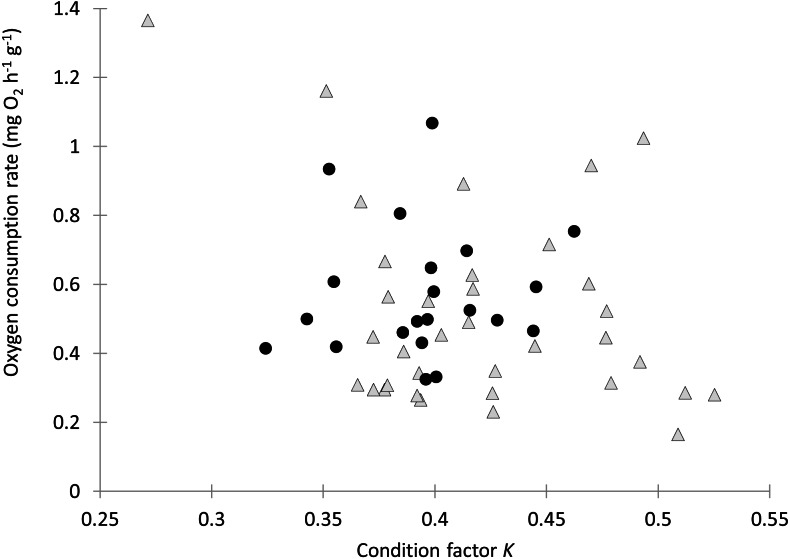
Oxygen consumption rate (mg O_2_ g^−1^ h^−1^) in relation to condition factor K of *Galaxias maculatus* parasitized (black circles) and unparasitized (gray triangles) by *Tylodelphys* sp.

**Figure 3:**
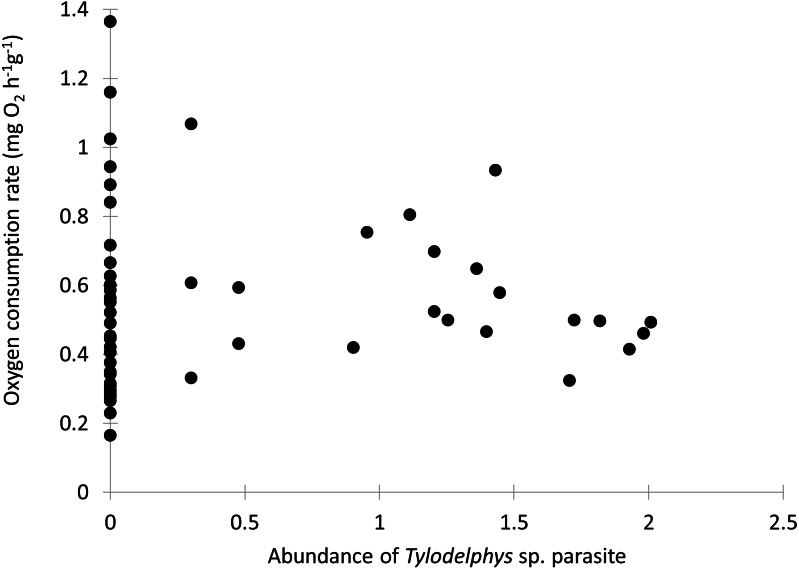
Oxygen consumption rate (mg O_2_ g^−1^ h^−1^) of *Galaxias maculatus* in relation to the abundance of *Tylodelphys* sp. in its cranial cavity (log_10_ (*x* + 1) transformed).

Individual fish varied in condition factors. No significant difference in Fulton’s condition factor (*K*) was found between parasitized and unparasitized fish (Mann–Whitney U test: *U* = 463, *P* = 0.054, *n* = 56). Similarly, no significant differences were found in the OC rates between parasitized and unparasitized fish (Mann–Whitney U test: *U* = 264, *P* = 0.951, *n* = 56) (for complete fish variables, see Table S2). Furthermore, the relationship between the OC rates (mg O_2_ g^−1^ h^−1^) and the Fulton’s condition factor of fish was not different between parasitized and unparasitized fish (ANCOVA: *F*_1, 53_ = 0.67, *P* = 0.417, Fig. 2). Finally, no significantly negative slope was found between the OC rates of infected fish (Theil test: *k* = 21.36, *P* = 0.147, *n* = 21; Fig. 3) or condition factor of fish (Spearman correlation: *r*_*s*_ = 0.188, *P* = 0.412) vs. the intensity of *Tylodelphys* sp.

When the behavioral variables were analysed individually, the median test showed significant differences only for the surface swimming level. Specifically, parasitized fish swam more often closer to the surface (median test: *Z* = 1.914, *P* = 0.027, Table 1). The frequency with which the fish swam in the middle (median test: *Z* = 1.367, *P* = 0.085, Table 1) and at the bottom (median test: *Z* = 0.820, *P* = 0.205, Table 1) of the aquarium was not significantly different between parasitized and unparasitized fish. The mobility of fish while kept in the undisturbed aquarium also did not differ between parasitized and unparasitized fish (median test: *Z* = 0.867, *P* = 0.193, Table 1). When facing a simulated predator attack, the unparasitized fish escaped (primary response) significantly more frequently compared to parasitized fish (median test: *Z* = 1.748, *P* = 0.040, Table 1). Furthermore, parasitized fish showed more freezing and immobility behaviors as a secondary response to predator stimuli compared to unparasitized fish (median test: *Z* = 1.963, *P* = 0.024, Table 1).

**Table 1:** Relative frequency (%) of *Galaxias maculatus* individuals found above and below the median (Me).

	P (*n* = 21)		NP (*n* = 35)		*p*
	>Me	<Me	>Me	<Me	
Swimming level S	67	33	40	60	*
M	38	62	57	43	n.s.
B	57	43	46	54	n.s.
Activity level	57	43	69	31	n.s.
Primary response to attack	52	48	67	33	*
Secondary response to attack	48	52	70	30	*

**Notes.**

In relation to oxygen consumption rates and each of the behavioural variables evaluated in fish parasitized (P) and unparasitized (NP) by *Tylodelphys* sp.; (*) significant differences; (n.s.) non-significant differences.

S Surface M middle B bottom

**Figure 4:**
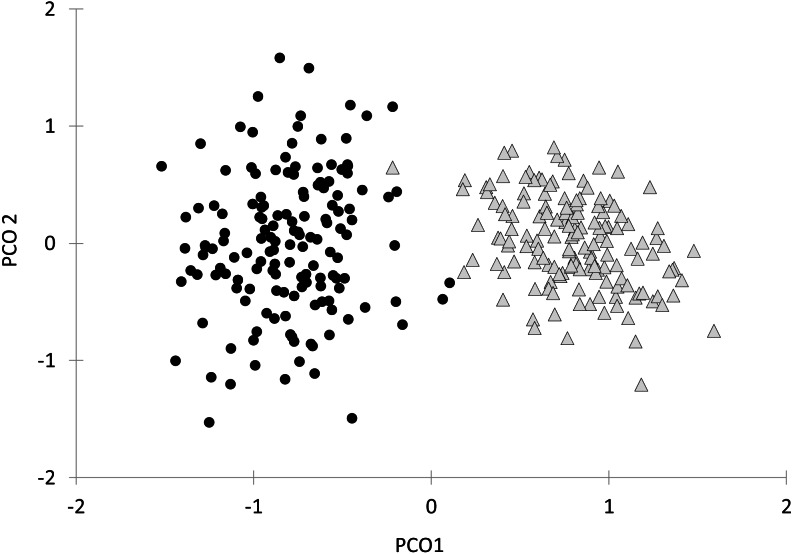
Principal Coordinate Analysis (PCoA) of the behavioural variables and oxygen consumption rates of *Galaxias maculatus*. Bootstrap data are displayed (*n* = 150 resamples). Fish parasitized by *Tylodelphys* sp. are indicated with circles and unparasitized fish with triangles. First (PCO1) and second (PCO2) axes are indicated.

Finally, the first axis of the Principal Coordinates Analysis of all behavioral variables together after bootstrapping the data explained 73.3% of the total variation (Fig. 4). Along this axis there was a clear separation of parasitized and non-parasitized fish, as indicated by PERMANOVA (Pseudo- *F*_1, 298_ = 176.89, *P* (perm) = 0.001, Table 2).

## Discussion

Differences in swimming behavior and escape responses of fish to a predator attack between parasitized and unparasitized fish are consistent with the hypothesis that *Tylodelphys* sp. could manipulate host behavior in a way that increases susceptibility to predation by the final host. These behavioral changes are observed without significantly affecting host condition, as measured by OC rates and Fulton’s condition factor. Manipulation without impairing host condition is consistent with findings from another brain-infecting trematode that infects fish brains in California (Lafferty & Morris, 1996; Shaw et al., 2009).

Parasitized fish often kept swimming close to the surface, and after a simulated predator attack they did not escape or move out of a resting position and had erratic swimming behavior, which may be evidence of parasitic manipulation (Gopko, Mikheev & Taskinen, 2017; Seppälä, Karvonen & Valtonen, 2008). Although we did not test predation rates directly, the surface swimming behavior could mean that fish are more visible and can be more easily caught by birds that do not dive deeply (Loot et al., 2002), suggesting an increased susceptibility to predation and therefore a greater probability of trophic transmission and reproductive success of the parasite (Barber, 2007; Barber, Hoare & Krause, 2000; Poulin, 2017; Thomas, Adamo & Moore, 2005). Other studies have shown a link between similar behavioral alterations and increased predation risk in wild fishes (Lafferty & Morris, 1996). Although we do not know the mechanisms behind this effect, a lack of response in another brain-infecting trematode is thought to be due to a change in neuroamines that reduce stress responses in fishes (Shaw et al., 2009). Presumably, infecting the brain makes it easier for a parasite to suppress or depress the escape response and reaction to a predator (Erickson, Dohi & Banks, 2012; Lafferty & Shaw, 2013; Shaw et al., 2009).

**Table 2:** Summary of PERMANOVA of the behavioural variables and oxygen consumption rates of *Galaxias maculatus*.

Source	d.f.	SS	MS	Pseudo-F	P
P-NP	1	193.6	196.33	176.89	0.001
Residuals	298	32.453	0.108		
Total	299	226.12			

**Notes.**

Bootstrapped data (*n* = 150 resamples) of fish parasitized (P) and unparasitized (NP) by *Tylodelphys* sp.; d.f., degrees of freedom; SS, sum of squares; MS, mean of squares; Pseudo-F distance based on pseudo-F statistic; P, probability value obtained using 999 permutations.

Although the fish came from two different localities (stagnant and flowing water), unpublished data (from previous laboratory observations) indicate that the fish were similar in feeding behaviors and phenotype (R. López-Rodríguez, 2019, personal observation). Furthermore, we would expect the abundance of predatory birds to be higher in a stagnant water body. Similarly, fish from stagnant water could be more sensitive to predator cues producing slight disturbance on the surface of water, and this disturbance could be masked in flowing water. However, in our case infected fish came from stagnant water and therefore their lack of response to predatory cue could be expected to be even stronger in flowing water. We also ensured similar size (age) of the fish and the time of acclimation (identical time of captivity, flow, aeration and temperature of the water) to allow the most possible unbiased comparisons. As such, we acknowledge the existence of confounding factors but we consider that the findings are a valid first approximation in this host-parasite system. These findings could be corroborated with future studies that consider larger samples sizes of fish from one geographic location and habitat and with experimentally infected fish when first intermediate host is described.

The lack of differences in the physiological variables tested (oxygen consumption rate and condition factor of the fish) most probably was caused by either the fact that the metabolic pathway does not correspond to the mechanism behind the behavioral changes of the hosts or the infection intensity does not correlate with the magnitude of the changes. Trematode metacercariae do not grow much in their fish host, for example compared with larval cestodes, and so their energetic demands are probably low. Furthermore, there might be selection on the parasite to minimize its negative effects on host condition to increase the chance that the fish dies via predation in the correct final host (Lafferty, 1997).

Joint multivariate analyses and use of resampling techniques allowed detection differences between parasitized and unparasitized fish (Anderson, 2001; Archer, Saltelli & Sobol, 1997), even when individual responses were relatively weak. Analyses of behavioral and physiological variables together are scarce in behavioral studies, and apart from few exceptions (Seppälä, Karvonen & Valtonen, 2008), behavioral and physiological variables are usually evaluated independently. Furthermore, behavioral studies often have few observations (small sample sizes) and therefore combination of multivariate analyses and resampling tools made it possible to integrate variables of different nature (e.g., behavioral and physiological data).

## Conclusions

Our results suggest that *Tylodelphys* sp. inhabiting the cranial cavity of *G. maculatus* could alter their behavior eventually causing an increase susceptibility to predation by birds. These results are consistent with the behavior-modification hypothesis and emphasize the importance of parasites for predator–prey interactions. These results should be corroborated in future studies with larger sample sizes of fish collected from the same population and with experimentally infected fish once first intermediate host is determined.

##  Supplemental Information

10.7717/peerj.11095/supp-1 Table S1 Endoparasites of *Galaxias maculatus* parasitized (P) and unparasitized (NP) with *Tylodelphys* sp Total number of parasites and taxa per group (bottomrows), and total number of parasites, prevalence (P%) and intensity of infection per taxa.Infection sites (is) and the development stages (D) of each taxon are indicated in footnote.

10.7717/peerj.11095/supp-2 Table S2 Average ± standard deviation of variables recorded in *Galaxias maculatus* parasitized (P) and unparasitized (NP) with *Tylodelphys* sp

## Additional Information and Declarations

### Field Study Permissions

The following information was supplied relating to field study approvals (i.e., approving body and any reference numbers): Field collections were approved by the Subsecretaría de Pesca y Acuicultura (R.EX No 4060).

### Data Availability

The following information was supplied regarding data availability: Data is available at Zenodo: Lopez-Rodriguez, Ruby. (2020). Dataset Tylodelphys sp. effects on *Galaxias maculatus* [Data set]. Zenodo. http://doi.org/10.5281/zenodo.4287644.
