# Supplementary material for: Effects of the cranial parasite Tylodelphys sp. on the behavior and physiology of puye Galaxias maculatus (Jenyns, 1842)
Source: PeerJ. 2021 Mar 22;9:e11095. doi: 10.7717/peerj.11095 (PMC7993012; doi:10.7717/peerj.11095)
Supplement: Table S1 — Total number of parasites and taxa per group (bottomrows), and total number of parasites, prevalence (P%) and intensity of infection per taxa.Infection sites (is) and the development stages (D) of each taxon are indicated in footnote. [file peerj-09-11095-s001.docx]

|  | P (n= 21) | | | NP (n = 35) | | |
| --- | --- | --- | --- | --- | --- | --- |
| Parasites taxa (is; D) | total | P (%) | intensity | total | P (%) | intensity |
| *Contracaecum*sp. (m, bc; L) | 10 | 28.6 | 1.7 | 1 | 2.9 | 1 |
| *Tylodelphys*sp. (cc; L) | 625 | 100 | 29.8 | 0 | 0 | 0 |
| *Posthodiplostomum* sp. (g, st; L) | 0 | 0 | 0 | 3 | 8.6 | 1 |
| *Achanthostomoidesapophalliformis* (li; M) | 11 | 23.8 | 2.2 | 41 | 74.3 | 1.6 |
| *Allocreadium pichi* (g; A) | 3 | 9.5 | 1.5 | 27 | 42.9 | 1.8 |
| Larva Cyclophyllida (g; L) | 8 | 19 | 2 | 0 | 0 | 0 |
| Total | 657 |  |  | 72 |  |  |
| N taxa | 5 |  |  | 4 |  |  |

is = infection site: g: gut, cc: cranial cavity, m: mesenteries, bc: body cavity, li: liver, st: stomach. D= development stage: A: adult, L: larva, M: metacercariae
