# Supplementary material for: Effects of the cranial parasite Tylodelphys sp. on the behavior and physiology of puye Galaxias maculatus (Jenyns, 1842)
Source: PeerJ. 2021 Mar 22;9:e11095. doi: 10.7717/peerj.11095 (PMC7993012; doi:10.7717/peerj.11095)
Supplement: Table S2 [file peerj-09-11095-s002.docx]

|  | P (n=21) | NP (n=35) |
| --- | --- | --- |
| Total length (cm) | 5,9 ± 0,73 | 6,4 ± 0,58 |
| Weight (g) | 0,8 ± 0,29 | 1,1 ± 0,38 |
| Condition factor (*K*) | 0,4 ± 0,03 | 0,4 ± 0,05 |
| Sexual ratio (M:F) | 1 : 1,25 | 1 : 1,12 |
| *Tylodelphys*sp. | 29,9 ± 32,0 | 0 |
| Oxygen consumption rate (mg O_2_ h^-1^g^-1^) | 0,517 ± 0,3 | 0,573 ± 0,2 |

(M:F: male:female)
